# Cell Tracking by Magnetic Particle Imaging: Methodology for Labeling THP-1 Monocytes with Magnetic Nanoparticles for Cellular Imaging

**DOI:** 10.3390/cells11182892

**Published:** 2022-09-16

**Authors:** Amani Remmo, Norbert Löwa, Olaf Kosch, Dietmar Eberbeck, Antje Ludwig, Lena Kampen, Cordula Grüttner, Frank Wiekhorst

**Affiliations:** 1 Physikalisch-Technische Bundesanstalt, Abbestraße 2-12, 10587 Berlin, Germany; 2 Charité, Center for Cardiovascular Research (CCR), Berlin, Hessische Straße 3-4, 10115 Berlin, Germany; 3 Micromod Partikeltechnologie GmbH, Schillingallee 68, 18057 Rostock, Germany

**Keywords:** magnetic nanoparticles, magnetic particle spectroscopy, magnetic particle imaging, cell tracking, THP-1 monocytes, microscopy

## Abstract

Magnetic particle imaging (MPI) is a noninvasive tomographic imaging modality for the quantitative visualization of magnetic nanoparticles (MNPs) with high temporal and spatial resolution. The general capability of MPI for cell tracking (e.g., monitoring living cells labeled with MNPs) has successfully been shown. MNPs in cell culture media are often subjected to structural and magnetic changes. In addition to the deteriorating reproducibility, this also complicates the systematic study of the relationship between the MNP properties and their cellular uptake for MPI. Here, we present a method for the preparation of magnetically labeled THP-1 (Tamm–Horsfall Protein-1) monocytes that are used in MPI cell tracking. The method development was performed using two different MPI tracers, which exhibited electrostatic and steric stabilizations, respectively. In the first step, the interaction between the MNPs and cell culture media was investigated and adjusted to ensure high structural and magnetic stability. Furthermore, the influences of the incubation time, MNP concentration used for cellular uptake, and individual preparation steps (e.g., the washing of cells) were systematically investigated. Finally, the success of the developed loading method was demonstrated by the MPI measurements. The presented systematic investigation of the factors that influence the MNP loading of cells will help to develop a reliable and reproducible method for MPI monocyte tracking for the early detection of inflammation in the future.

## 1. Introduction

Magnetic particle imaging (MPI) has recently been introduced as a preclinical diagnostic tool for the visualization of the spatial distribution of magnetic nanoparticles (MNPs). MPI allows for the specific detection of MNPs, without any background signals from biological tissues. The suitability of MPI cell tracking (i.e., the monitoring of living cells labeled with MNPs) has been successfully demonstrated [1]. Cell imaging is the noninvasive and repeated imaging of specific cells and cell processes in living organisms [2], and it is of high clinical relevance (e.g., for the early detection of inflammatory processes) [3]. Inflammation is associated with a variety of diseases, such as atherosclerosis, cancer, aortic aneurysms, and ischemic heart disease [4,5]. A specific approach for early inflammation detection is the tracking of monocytes, which play a key role in the inflammatory response. These monocytes patrol the vasculature, are activated by signaling agents, and then migrate into the inflamed tissue. Thus, the imaging of monocytes is an advantageous approach for the early detection of inflammatory processes [5]. Additionally, cell imaging can also be used to investigate the migration and fate of these cells, and their biological role in the organism. This helps to better understand inflammatory processes and develop novel ways to positively influence them [6,7].

Several methods are available for noninvasive cell imaging, each using specific signal generators and requiring the cells to be labeled prior to use. Because optical methods are often limited due to the low penetration depth of light, and examination methods with radioactive markers are restricted in terms of the dose and repeatability, magnetic resonance imaging (MRI) is currently the method of choice for cell tracking [8]. To produce a sufficient MRI contrast above the tissue background, the cells are loaded with MNPs (as in MPI) to cause a local magnetic field disturbance that locally affects the measured H-proton signal in MRI [9]. The individual MNP-loaded cells thus generate a characteristic signal pattern and can be localized [10]. Furthermore, it was shown that these effects of MNP-loaded cells on MR signaling can also be used to discriminate between free and cell-bound MNPs [11,12].

However, because MNPs only indirectly influence the MRI signal, quantitative evaluation is difficult in many cases (e.g., cell clustering or changing MNP arrangement) [13,14]. The greatest challenge in labeling cells with MNPs is to achieve a sufficiently high MNP loading while maintaining the vital and functional properties of the cell (e.g., cell division, differentiation). This is the basic prerequisite for imaging cells with high sensitivity, and for studying their behavior in an organism. Due to the phagocytic properties of macrophages and monocytes, loading with MNPs can occur directly, but it is still determined by the colloidal state and surface properties of the MNPs [15]. The lack of colloidal stability of MNPs may lead to an undesired MNP overestimation of the cellular uptake and erroneous cell tracking [16]. MPI, as a noninvasive radiation-free alternative with a high penetration depth and high temporal resolution, provides significant added value compared with the established imaging methods [17]. Additionally, MPI provides a quantitative procedure for specific MNP detection, in which the measured signal contains information about the quantity, position, type, and state of the MNPs [1]. Largely unexplored is the influence of the individual preparation steps and employed substances on the loading of cells with MNPs. This is particularly relevant to the interaction between MNPs and biological media before and during cellular uptake.

In this work, we present a developed method to achieve the reproducible labeling of THP-1 monocytes for MPI imaging. The structural and magnetic changes in the MNPs were considered before and during cellular uptake, as well as the loading state and the viability of the loaded cells. Finally, we demonstrated the success of cell loading using MPI measurements on intracellular MPI tracers.

## 2. Materials and Methods

### 2.1. Magnetic Nanoparticles (MNPs)

We used two commercial MNP systems: Synomag^®^ (LOT: 12121103-01, Syn-COOH) and Synomag^®^-D (LOT: 12821104-01, SynD-PEG) (micromod Partikeltechnologie GmbH, Rostock, Germany). Syn-COOH has already been successfully employed for MPI cell imaging [1], while SynD-PEG is often used in MPI as a long-term stable MNP system [18]. The two MNP systems have the same core diameter (see Table 1), but they deviate in the coating and functional groups. Details on the origin and structural and magnetic properties of the MNPs are summarized in Table 1.

### 2.2. Cell Line: THP-1 Monocytes

The experiments are performed with THP-1 monocytes. THP-1 monocytes are human tumor cells that are isolated from patients with acute monocytic leukemia. The THP-1 monocytes were passaged every 3–4 d. Passages from 5 to 15 were used for the experiments. For the estimation of the effect of the Syn-COOH loading on the cell proliferation, THP-1 cells were loaded with 0.5 mmol/L Syn-COOH for 10 min in PBS, washed twice with PBS, and seeded at 0.4 × 10^6^ cells in 1 mL medium supplemented with 10% FCS in 24-well plates. The cells were cultured for an additional 72 h. The cell counts were n_1_ at 24 h, and n_2_ at 72 h, after seeding. The doubling time (*t*_D_) was calculated as *t*_D_ = 48 h·ln(2)/ln(n_2_/n_1_). The cells were purchased from ATCC^®^ (Wesel, Germany). The cultivation was performed in a 75 cm^2^ cell culture flask in a suspension of RPMI medium 1640 (Invitrogen, Dreieich, Germany) in an incubator (37 °C, 5% CO_2_). The culture medium was supplemented with 10% fetal calf serum (FCS) (Biochrom, Berlin, Germany) and 1% penicillin-streptomycin (Invitrogen, Dreieich, Germany). An exchange of the medium took place every 2–3 days. After reaching a density of 5 × 10^5^ cells/mL, the suspended THP-1 monocytes were diluted with a new medium at a ratio of 1:3. For the visualization of the loaded THP-1 monocytes, light microscopy images were made with a fluorescence microscope (BZ-X81, Keyence, Leipzig, Germany). The loaded THP-1 monocytes were spotted on a glass slide, and the iron was visualized by Prussian blue staining (2% potassium ferrocyanide (Sigma-Aldrich, Darmstadt, Germany)) in 1% hydrochloride acid (Merck, Darmstadt Germany), followed by counterstain with Nuclear Fast Red (Carl Roth, Karlsruhe, Germany).

### 2.3. Biological Media

All chemicals used in this study were of analytical reagent grade. The media deionized water (ddH2O), phosphate-buffered saline (PBS) (Gibco, Dreieich, Germany), fetal calf serum (FCS) (Biochrom, Berlin, Germany), and Roswell Park Memorial Institute 1640 medium (RPMI 1640 Medium, Gibco, Dreieich, Germany) were used in this work.

### 2.4. Field-Flow Fractionation (F3)

To evaluate the MNP behavior in different biological media prior to cell contact, we performed centrifugal flow-field fractionations (CF3, Postnova Analytics GmbH, Landsberg am Lech, Germany). In CF3, the sample is transported within the main flow through a thin channel (350 µm height) [19]. A centrifugal force drives the particles towards the channel wall in a rotating disk. By this, a temporal separation of the sample according to the particle mass is achieved (i.e., smaller MNPs are eluted earlier from the separation channel than larger ones). A rotational speed of *v*_rot_ = 4900 1/min, ddH_2_O as carrier liquid, and an MNP concentration of *c*(Fe) = 5 mmol/L were used for the separation. The fractions were afterwards magnetically and structurally characterized using the online detector array of CF3 consisting of an MPS (MPS-3, Bruker BioSpin, Rheinstetten, Germany), a device for dynamic light scattering (DLS) (Zetasizer NanoZS, Malvern, UK), and an ultraviolet/visible (UV/Vis) spectrophotometer (Postnova Analytics, Landsberg am Lech, Germany) [19]. The detectors are described in the following paragraphs (2.5–2.7).

### 2.5. Ultraviolet/Visible Spectrophotometer (UV/Vis)

A UV/Vis spectrophotometer (PN 3211, Postnova Analytics GmbH, Landsberg am Lech, Germany) was used to measure the light absorption of the MNPs in different media after separating them with CF3. Here, the UV/Vis is directly coupled to the CF3 channel outlet so that the MNPs in the media can flow through the UV/Vis flow cell. This flow cell is made of quartz glass and comprises a measuring volume of 12 µL. In this work, a wavelength of *λ* = 350 nm was used.

### 2.6. Magnetic Particle Spectroscopy (MPS)

To magnetically characterize the MNPs in different media and the MNP-loaded cells, we used MPS that measures the nonlinear magnetic susceptibility of MNP samples. The measurements were performed at body temperature (*T* = 37 °C) using a commercial MPS device (MPS-3, Bruker, Rheinstetten, Germany), operated at a sinusoidal excitation field (amplitude up to *B* = 25 mT, fixed frequency of *f*_0_ = 25 kHz). For using MPS in the online detector array with CF3, a flow cell was used, where the MNPs move in a capillary through the MPS detector coil (*B* = 25 mT) [20]. The MPS flow cell consists of a 3D-printed plastic holder for the reproducible fixation and positioning of the capillary. The capillary is made of fluoroethylene propylene, with an inner diameter of 0.8 mm, and an outer diameter of 1.59 mm. The active measuring volume in the detector coil is 33.5 µL [20]. MPS measurements in batch mode were performed with a sample holder, where an MNP-filled PCR tube (fast reaction tubes with cap, Appl. Biosystems, Waltham, MA, USA) was placed in the sample holder and carried out at *B* = 12 mT. An averaging time of 10 s was chosen to improve the signal-to-noise ratio. To compare the different MNP systems, the specific amplitude (*A*_3_*) is calculated (e.g., the *A*3 amplitude normalized to the absolute iron mass of the sample). The third harmonic amplitude (*A*3) is directly proportional to the absolute MNP content and can be used for MNP quantification. The MPS noise level was determined according to the guidance of the International Union of Pure and Applied Chemistry (IUPAC) as the mean + 3 ×standard deviation of the empty-sample-holder measurements. Furthermore, the slope of the MPS spectrum was parametrized by the ratio of the fifth (*A*_5_) and third (*A*_3_) harmonic amplitudes. The *A*_5_/*A*_3_ ratio reflects (changes in) the dynamic magnetic behavior of MNPs, and it is used as an indicator for the resolution of an MNP sample in MPI.

### 2.7. Dynamic Light Scattering (DLS)

For the measurements of the hydrodynamic size of the MNPs, a particle sizer at room temperature (*T* = 20 °C) (Zetasizer NanoZS, Malvern Instruments, Cambridge, UK), equipped with a He–Ne laser (*λ* = 632.8 nm), was used. For using the DLS in the online detector array, a flow cell with a volume of 70 µL was used. The experiments were conducted in backscattering mode at a scattering angle of 173°. For the DLS batch measurements, the samples were placed in a square 10 × 10 mm disposable polystyrene cuvette. The hydrodynamic diameter (*d*_hyd_) (spherical noninteracting particles assumed) was obtained from the diffusion coefficient using the Stokes–Einstein relation [21]. Furthermore, the device was used to measure the surface charge (zeta potential (*ζ*)) of both MNPs at a pH of 7.

### 2.8. Spectrophotometric Determination of Iron

For the quantification of the iron mass in the MNP-loaded cells, the spectrophotometric determination of iron with 1,10-phenanthroline was used as an alternative to MPS-based MNP quantification (see Section 2.3). For this, we used the SpectraMAX Plus 384 (Molecular Devices, Wokingham, UK). The detailed approach of the iron determination with 1,10-phenanthroline is described elsewhere [22]. In short, the color intensity of a solution is determined to infer the concentration present as iron +II reacts with 1,10-phenanthroline and forms a colored complex ion. A calibration curve (absorbance over concentration) is constructed for the iron +II, and the concentration of the unknown sample is determined.

### 2.9. Small-Angle X-ray Scattering (SAXS)

The core diameters of the MNPs were determined by an analysis of the SAXS data measured with an XRD device (SAXSpace system, Anton Paar, Buchs, Switzerland). It allows for the core size determination of MNPs in a diameter range between 1 nm and 300 nm. For the measurements, the MNPs were diluted to an iron concentration of 5 mmol/L. The dispersion was injected into a flow cell, and the scattering pattern was measured. After the absorption correction, the background scatter contributions of the flow cell and dispersion medium (here, ddH_2_O) were subtracted from the measurement data. To determine the core size distribution, the model of lognormally distributed spherical particles was fitted to the corrected data [23]. The smearing of the data due to the line collimation of the primary beam was taking into account by smearing the model with the measured profile of the beam. The cores of the Synomag^®^ MNPs consist of multiple cores. Therefore, one may denote them as multicore-MNPs (MCMNPs), or nanoflowers [24]. The investigations of Bender et al. showed that MCMNPs are quite compact and nearly spherical. Therefore, we considered the application of the single-core model valid for the estimation of the mean outer contour of the MCMNPs (see Table 1). For the analysis, we used only the data at *q* < 0.5 nm^−1^ because the small-scale inner structure and surface roughness of the MCMNPs are not covered by the applied model.

### 2.10. Magnetic Particle Imaging (MPI)

We used a commercial preclinical MPI scanner (MPI 25/20 FF Bruker BioSpin, Ettlingen, Germany) to analyze the imaging performance of the loaded cells. This system is a field-free-point (FFP) scanner that is based on the system-function approach for image reconstruction. Field gradients of 2.5 T/m in z-, and 1.25 T/m in x and y, were used for the spatial coding. For the measurement, the FFP is moved through the FOV on a closed 3D Lissajous trajectory by applying three orthogonal driving fields of a 12 mT amplitude, oscillating at slightly different excitation frequencies (2.5 MHz divided by 102/96/99 in the x-/y-/z-directions, respectively), and the MNP responses are recorded. For the image reconstruction, the acquisition of the corresponding system function (SF) has to be performed in a separate calibration measurement for each MNP type. For this purpose, a point-like reference sample of MNPs (volume of about 30 µL) is positioned by a robot at all selected grid positions within the FOV of 25.5 mm × 25.5 mm × 3 mm. For this purpose, a point-like reference sample of MNPs (volume of about 30 µL) is positioned by a robot at all selected grid positions within the FOV of 25.5 mm × 25.5 mm × 16.5 mm, and the resulting MNP responses during the FFP trajectory cycles are recorded. After the background subtraction, all components with an SNR threshold greater than five were used. Images were obtained using the Kaczmarz algorithm for regularization, with 20 iterations and a relative regularization parameter of 0.001. To increase the overall sensitivity by +12 dB, we used the recently developed separate receive coil [25], which gradiometrically minimizes the direct coupling of strong excitation fields into the receive chain of the MPI scanner. For the quantitative evaluation, the amount of MNPs in the cell samples was determined relative to the amount of the reference sample that was used for the SF acquisition. Two different methods of summing up the voxels were performed: 1. the total estimate, which adds up the contributions of every single voxel with a content higher zero; 2. the segmented estimate, which selects the voxel with the highest iron content to which the contents of all the neighboring voxels with iron contents higher than 10% of the maximum value are added.

## 3. Results and Discussions

### 3.1. Basic Characterization of the MNP

The properties of the MNP suspensions are summarized in Table 1. Assessing the core diameter determined with SAXS, we observed that both MNP samples had similar core diameters of about 15 nm (Table 1). In contrast, the hydrodynamic diameters of both MNP samples were significantly different, as the *d*_hyd_ of Syn-COOH was half that of SynD-PEG, with *d*_hyd_ = 23.4 nm and *d*_hyd_ = 47.7 nm, respectively. The reason is the significantly thicker double layer of polyethylene glycol (PEG) and dextran of SynD-PEG. The zeta potential measurements at a pH of 7 showed that Syn-COOH has a more negative charge (*ζ* = −13.8 mV) compared with SynD-PEG (*ζ =* −4.6 mV).

### 3.2. MNP–Media Interactions before Cell Contact

**Figure 1:**
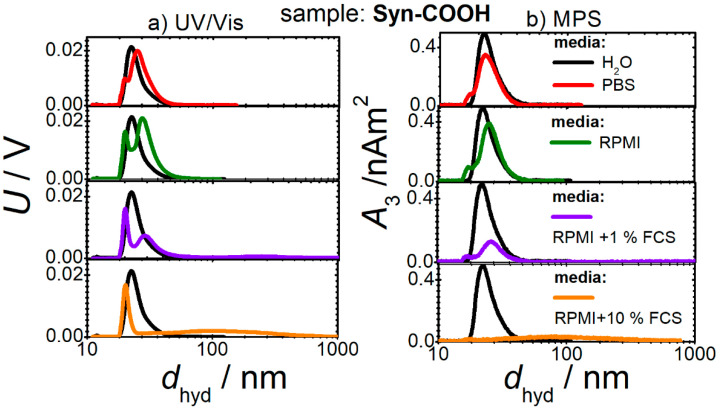
Iron concentration (*c*(Fe)) (obtained from (**a**) UV/Vis) and magnetic amplitude (*A*_3_) (measured by (**b**) MPS) as a function of hydrodynamic diameter (*d*_hyd_) obtained (by DLS) during CF3 separations of Syn-COOH in four different media.

The magnetic behavior of MNPs might be changed when they are suspended in different media [26]. To ensure reproducible and controlled MNP cell loading for MPI, the interaction between the media and MNPs before cell contact was investigated by CF3. The CF3 sorts the MNPs according to their masses. The direct online measurement of the time-increasing hydrodynamic size, as well as the UV/Vis (Figure 1a) and MPS (Figure 1b) signals, allow these quantities to be contrasted for an accurate analysis of the structural and magnetic changes. This method is superior to the single-batch methods, and especially for the analysis of broad distributions. The UV/Vis signal as a function of the size and reflects the size distribution of the investigated samples, whereas the size-dependent MPS signal has the advantage of representing only MNP changes, and no additionally contained macromolecular substances. The Syn-COOH in PBS shows a slight peak broadening in UV/Vis (Figure 1a) (i.e., a broadening of the size distribution). For the MNPs in RPMI, a peak splitting and slight peak shift to large diameters occurs. Interestingly, the MPS evaluation (Figure 1b) showed that the first peak did not contain any MNPs. In RPMI with added FCS, the width of the MNP size distribution increases even more, and large aggregates (about 100 nm) are present at 10% FCS, which can be detected by both UV/Vis and MPS.

**Figure 2:**
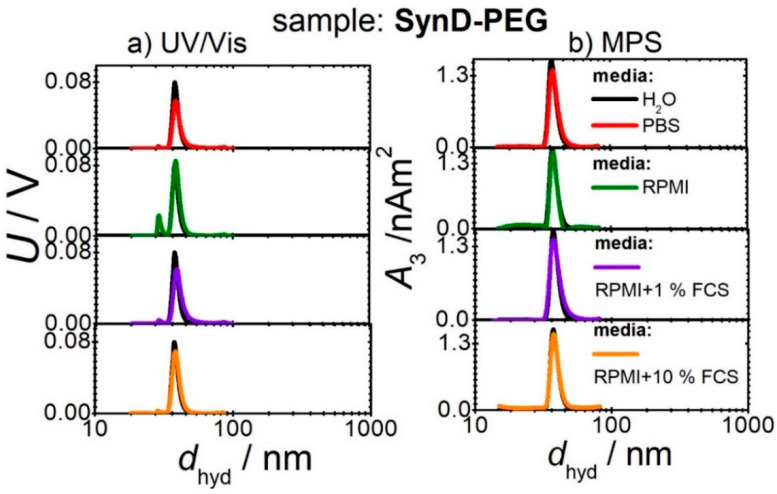
Iron concentration (*c*(Fe)) (obtained from (**a**) UV/Vis) and magnetic amplitude (*A*_3_) (measured by (**b**) MPS) as a function of hydrodynamic diameter (*d*_hyd_) obtained (by DLS) during CF3 separations of SynD-PEG in four different media.

In comparison, no significant changes in the size distribution are seen for SynD-PEG in all four media (Figure 2). SynD-PEG shows higher stability in the studied media compared with Syn-COOH, as no significant change in the size distribution could be detected.

Nevertheless, in the following cell experiments, FCS is not used in the cell culture medium during cell loading. The exclusive use of PBS is preferred. Because the culture medium of THP-1 monocytes also contains FCS, the cells are washed prior to the cellular MNP uptake to avoid the unwanted aggregation of MNPs.

### 3.3. MNP–Media Interactions during Cell Contact

#### 3.3.1. Media Check

**Figure 3:**
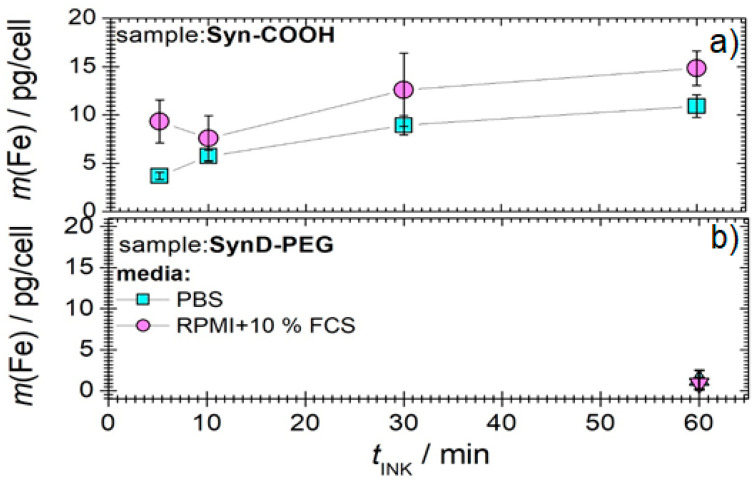
The iron mass per cell in 10^6^ THP-1 monocytes determined with the spectrometer after the incubation times of 5, 10, 30, and 60 min for both MNP (**a**) Syn-COOH and (**b**) SynD-PEG in PBS and RPMI with 10% FCS. The uncertainty represents the standard deviation from three repeated measurements.

**Figure 4:**
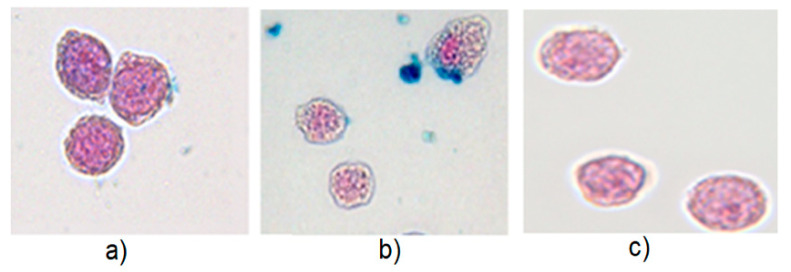
Light microscopy images of THP-1 monocytes after Prussian blue staining of iron Nuclear Fast Red staining of nuclei. Cells were incubated with (**a**) Syn-COOH in PBS, (**b**) Syn-COOH in RPMI with 10% FCS, and (**c**) SynD-PEG in PBS. The cellular uptake proceeds at an iron concentration of 0.5 mmol/L and incubation time of 15 min.

The cellular uptake of MNPs in the presence of various media was quantified using the spectrophotometric determination of iron (Figure 3), and it was examined by light microscopy (Figure 4). Figure 3 shows the iron mass per cell as a function of the incubation time for both MNP types in PBS and RPMI with 10% FCS. Syn-COOH shows an increasing iron mass per cell with the increasing incubation time (Figure 3a). Here, the presence of PBS seems to promote the amount of MNP uptake. The standard deviation (of three replicates) of the iron amount per cell in RPMI with 10% FCS is lar+-compared with PBS. Here, the medium RPMI with 10% FCS showed a higher tendency to form aggregates. In contrast, SynD-PEG showed no cellular uptake in either medium after 5, 10, and 30 min. After 60 min of incubation, a small amount of iron (0.5 pg/cell) could be detected. Compared with Syn-COOH, after 60 min, an iron content of around 10 pg/cell was detected. Here, the stable PEG–dextran coating seemed to prevent the cellular uptake.

In Figure 4, the microscopy images of Syn-COOH (media: PBS and RPMI with 10% FCS) and SynD-PEG (medium: PBS) are shown. The blue staining around the THP-1 monocytes and in the space between the cells are MNPs that have formed large aggregates.

Figure 4 shows that SynD-PEG was not taken up by the THP-1 monocytes. Moreover, the THP-1 monocytes in RPMI with 10% FCS showed a higher tendency to form aggregates. This is confirmed by Figure 1 and Figure 3a, where aggregation was also observed for MNPs interacting with FCS. For the reproducible and controlled cellular uptake of MNPs for MPI, FCS should not be used, whereas PBS is advantageous for cellular MNP uptake.

#### 3.3.2. Incubation Time and Iron Concentration

**Figure 5:**
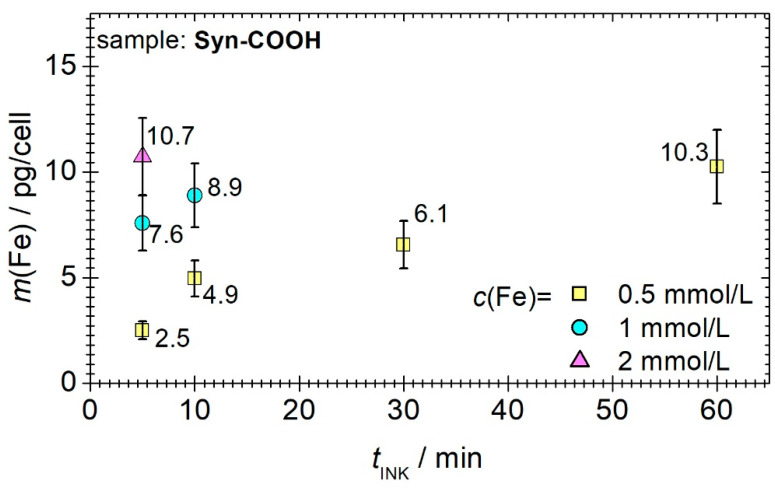
The iron mass determined by MPS at *B* = 12 mT as a function of the incubation time for three different iron concentrations of Syn-COOH (*c*(Fe) = 0.5, 1, and 2 mmol/L) in 10^6^ THP-1 monocytes. The uncertainty was determined from an 8-fold repeat of the measurement. For iron concentrations higher than 0.5 mmol/L, only *t*_INK_ for 5 and 10 min was reproducibly determined due to higher cell stress and cell death.

The cellular uptake of the MNPs was quantified after different incubation times (*t*_INK_ = 5, 10, 30, and 60 min), and at three different iron concentrations (*c*(Fe) = 0.5, 1 and 2 mmol/L), using MPS (see Figure 5). We found that both the iron masses per cell increased with the incubation time. Unfortunately, the variation in the iron masses per cell also increased. To avoid this effect, higher iron concentrations were used with the aim of reducing the incubation times. On the one hand, it was measured that a cell loading of about 10 pg/cell can be achieved six times faster if a double iron concentration is used. On the other hand, too high iron concentrations resulted in cell death, and therefore the loading state could not be determined for 1 mmol/L and 2 mmol/L for incubation times longer than 10 min. Ultimately, an iron concentration of 0.5 mmol/L and incubation time of 10 min were used because this iron concentration and incubation time had no effect on the morphology and population doubling time of the THP1 monocytes (Syn-COOH *t_D_* = 42.0 ± 1.03 h vs. control *t*_D_ = 42.7 ±1.57 h, *N* = 3), indicating that Syn-COOH is not toxic at the intracellular concentration reached.

#### 3.3.3. Washing of MNP-Loaded Cells

**Figure 6:**
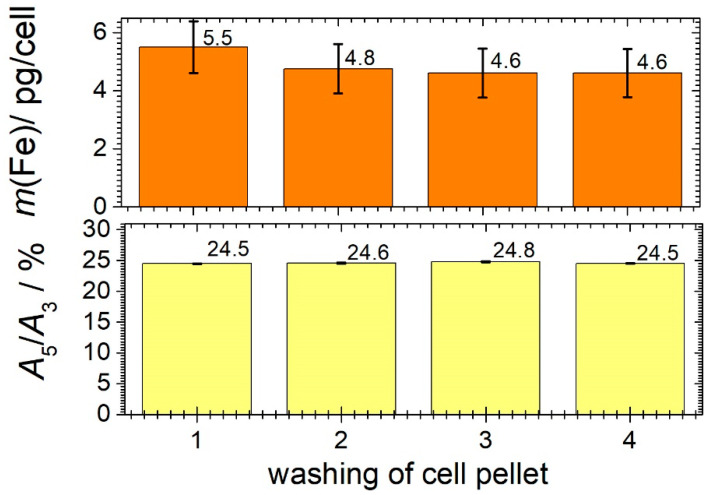
The iron mass determined by MPS at *B* = 12 mT for repeated washing of 10^6^ THP-1 monocytes loaded with Syn-COOH (*c*(Fe) = 0.5 mmol/L) after an incubation time of 10 min. Uncertainty was determined by repeating the wash series eight times. The numbers on each bar denote the corresponding values of *m*(Fe) and *A*_5_/*A*_3_.

In the previous measurements, aggregate formation was observed during cell loading. To avoid MNPs still being loosely adhered to the cell surface after cell loading, which could detach from the cell during the subsequent MPI cell tracking, the cells are washed. Therefore, we investigated how often the washing steps need to be repeated to remove loosely bound MNPs on the cell surface. To wash the loaded cells, the cells were resuspended by the addition of PBS. Subsequently, the loaded cells were separated into cell pellet and supernatant by centrifugation. The MNP iron mass in the remaining cell pellet was then quantified by MPS. The upper graph in Figure 6 shows the MNP iron mass in the cell pellet as a function of the number of washing steps. The lower graph shows the ratio *A*_5_/*A*_3_ indicating possible changes in the dynamic magnetization behavior of the MNPs. At constant *A*_5_/*A*_3_ values, the quantification of the MNP iron in the cells is highly accurate.

After washing the cells three times, the iron mass decreased by about 20%. The *A*_5_/*A*_3_ ratio is not affected by washing, which ensures an accurate MNP iron quantification. To avoid the cell viability being affected by washing, but also to remove loose MNPs on the cell surface, the cells should be washed at least twice after MNP loading.

#### 3.3.4. Sensitivity, Linearity, and Limit of Detection

**Figure 7:**
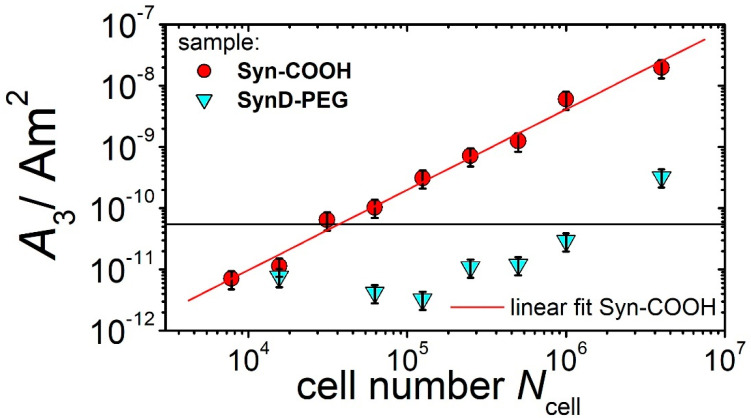
MPS signal amplitude (*A*_3_) as a function of cell number (*N*_cell_) (from 7800 to 4 × 10^6^ THP−1 monocytes), loaded with Syn-COOH (red circles) and SynD-PEG (blue triangles), and measured at *B* = 12 mT. The horizontal line represents with 5.5 × 10^−11^ Am^2^ the MPS noise level (mean + threefold standard deviation of 40 blank measurements of *A*_3_).

Finally, we analytically evaluated the quantitative magnetic detection of the labeled cells, including assessments of the linearity, sensitivity, and limit of detection (LOD), using MPS at *B* = 12 mT. Our linearity assessments involved creating and measuring dilution series of cells labeled with Syn-COOH and SynD-PEG in PBS, starting with 4 × 10^6^ cells. Figure 7 shows the MPS amplitude (*A*_3_) as a function of the cell number. For the Syn-COOH-labeled THP-1 monocytes, we found a linear scaling of the *A*_3_ (correlation coefficient: *R*^2^ = 0.986), and a sensitivity of *A*_3_* = 2.03 fAm^2^/cell. In contrast, for the SynD-PEG-loaded cells, only the signal amplitude (*A*_3_) of the initial sample was above the MPS noise level (5.5 × 10^−11^ Am^2^), which is why the linearity and sensitivity could not be determined. This is mainly due to the significantly lower loading of MNPs in the cells (SynD-PEG: 0.07 pg/cell, Syn-COOH: 13 pg/cell). Consequently, the limit of detection (LOD) for the SynD-PEG-loaded cells was also significantly higher than that of the Syn-COOH-loaded cells, with *N*_cell_ = 1.5 × 10^6^ and *N*_cell_ = 3 × 10^4^, respectively. It is expected that the LOD in MPI will be higher than in MPS due to the 100-fold higher MPI noise level [27].

### 3.4. MPI Visualization

**Figure 8:**
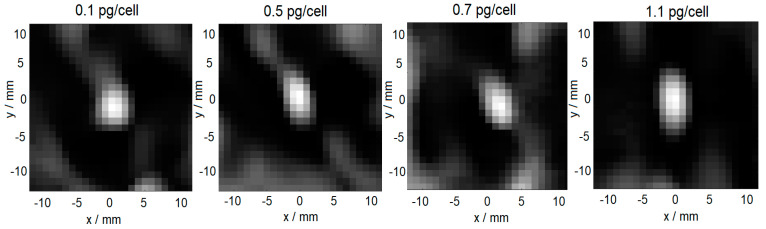
Reconstructed MPI images of THP−1 monocytes loaded with different amounts of Syn-COOH (*m*(Fe) = from 0.05 to 1.1 pg/cell).

Based on the previous results, we decided to use 10^6^ THP-1 monocytes loaded with different amounts of Syn-COOH in PBS (from 0.1 pg/cell up to 1.1 pg/cell) for the demonstration of MPI cell tracking. For the reconstruction of the MPI image, an SF was recorded using a point-like sample of a high concentration. Due to the low concentration of the cells, the sample Syn-COOH (*c*(Fe) = 50 mmol/L, *V* = 30 µL, in a liquid state was used. Starting from *m*(Fe) = 0.1 pg/cells up to 1.1 pg/cells, the MPI was capable of visualizing the THP-1 monocytes (see Figure 8).

The quantified MNP iron scaled linearly with the increasing cell-loading state. However, among other factors, the MPI signal of MNPs depends on their environmental conditions and aggregation state. Consequently, the SF sample should reflect the state of the MNPs in the object of investigation (MNPs in cells). Here, the SF with liquid Syn-COOH without THP-1 monocytes (*A*_5_/*A*_3_ = 12.3%) was used. In comparison, loaded THP-1 monocytes with Syn-COOH have an *A*_5_/*A*_3_ of 25%. This indicates that the SF of liquid Syn-COOH does not reflect the state of the MNPs in the cells, and it should be improved in the future to improve the image reconstruction and quantification of MNP-labeled cells.

## 4. Conclusions

The reproducible and controlled loading of THP-1 monocytes for MPI requires a systematic investigation of the variables that influence the loading process (media, incubation time, MNP concentration, etc.). It was shown that PBS is one of the biological media that least affects MNPs before and during cell loading. We further found that SynD-PEG, with its stabilizing PEG–dextran coating, is not taken up by cells (except for the low cellular uptake after an incubation time of 60 min and with the 4 ×10^6^ THP-1 monocytes). In contrast, the cellular uptake of Syn-COOH with an electrostatic stabilizing shell was significantly stronger. For the optimal quantification of THP-1 monocytes with MPS and MPI, the sufficient loading of MNPs is required. By studying the influence of the MNP concentration and incubation time, and considering the cell viability, we were able to determine the optimal conditions for loading THP-1 cells (*t*_INK_ = 10 min, *c*(Fe) = 0.5 mmol/L). To remove loosely bound MNPs on the cell surface after cell loading, the loaded cells should be washed at least two times with PBS. Based on the preliminary investigations [1], the THP-1 monocytes loaded with Syn-COOH could successfully be visualized by MPI, even at very low loading states (0.1 pg/cell). In this first MPI application, liquid Syn-COOH was used as the SF sample to enable the MPI image reconstruction. However, Syn-COOH does not reflect the state of MNPs in THP-1 monocytes, and therefore, future studies should include the selection of an appropriate SF sample for loaded cells that ensures reliable MPI image reconstruction. The use of a separation and online detectors array enabled a comprehensive, reliable, and rapid structural and magnetic analysis of MNPs in various cell culture media. Using the highly sensitive and fast MPS method in batch mode, combinations of cell types and MNP systems were elegantly and directly investigated and evaluated for their MPI cell-tracking suitability. This measurement infrastructure represents a powerful and indispensable tool for the development of reliable and controlled cell tracking using MPI.

## Figures and Tables

**Table 1:** Summary of the two different MNP systems used in this work. Given are the name and ID used in the graphs and text, together with the name of the supplier and coating of the MNP. Furthermore, the hydrodynamic diameter (*d*_hyd_ (z-average)), polydispersity index (PDI), and zeta potential (*ζ*) at a pH of 7, obtained by DLS and the characteristic MPS parameters (*A*_3_*) (*A*_3_ normalized to iron amount, and shape parameter *A*_5_/*A*_3_ as determined for the stock suspension) are presented. The MPS measurements were performed at *B* = 12 mT. The MPS noise level for the iron amount (determined from the magnetic moment amplitude with 5 × 10^−11^ Am^2^) was determined as the iron mass of an MNP sample corresponding to where the MPS noise level was defined as the mean + 3xstandard deviation of *A*_3_ resulting from 40 blank measurements after correction for the empty sample background.

ID	Supplier	Coating	MPS		DLS			SAXS	
			*A*_3_* (Am^2^/kg(Fe))	*A*_5_/*A*_3_ (%)	*d*_hyd_ (nm)	PDI	*ζ* at pH = 7 (mV)	*d*_c,SAXS_(nm)	*σ* (nm)
Syn-COOH	Micromod	Citrate	3.4 ± 0.05	12.6 ± 0.07	23.4 ± 6.2	0.06 ± 0.01	−13.8 ± 2.1	13.5 ± 0.2	0.27 ± 0.01
SynD-PEG	Micromod	PEG–dextran	6.1 ± 0.03	22.1 ± 0.05	47.7 ± 17.2	0.1 ± 0.03	−4.6 ± 1.2	15.0 ± 0.3	0.31 ± 0.01

## Data Availability

Not applicable.
